# Anxiety, Burnout and Depression, Psychological Well-Being as Predictor of Healthcare Professionals’ Turnover during the COVID-19 Pandemic: Study in a Pandemic Hospital

**DOI:** 10.3390/healthcare10030525

**Published:** 2022-03-14

**Authors:** Ayhan Tabur, Safa Elkefi, Abdurrahim Emhan, Cengiz Mengenci, Yasin Bez, Onur Asan

**Affiliations:** 1 Emergency Department, Gazi Yaşargil Training and Research Hospital, Diyarbakir 21070, Turkey; ayhan.tabur@saglik.gov.tr; 2 Stevens Institute of Technology, School of Systems and Enterprises, Hoboken, NJ 07030, USA; selkefi@stevens.edu; 3 Collage of Business Administration, University of Central Florida, Orlando, FL 32816, USA; abdurrahim.emhan@ucf.edu; 4 Department of Quality, Bower Hospital, Diyarbakir 21100, Turkey; cengizmengenci@yahoo.com; 5 School of Medicine, University of Miami, Miami, FL 32136, USA; yasin.bez@jhsmiami.org

**Keywords:** anxiety, depression, burnout, turnover, COVID-19, Turkey, healthcare, psychological health

## Abstract

Turnover of healthcare professionals’ is a rapidly growing human resource issue that affects healthcare systems. During the COVID-19 pandemic, healthcare professionals have faced stressful situations that have negatively impacted their psychological health. In this study, we explored impacts of the emotional wellbeing of healthcare professionals on their intention to quit their jobs. A cross-sectional survey design was used for this study. The respondents were selected based on simple random sampling. In total, 345 questionaries were returned and used for the analysis. Respondents were healthcare professionals (nurses, doctors, midwives, technicians, etc.) working in a pandemic hospital in Turkey. A multivariable logistic regression model was used to predict the emotions that encouraged the respondents to intend to quit their jobs. Emotions including anxiety, burnout, and depression were measured using validated scales. We found that the COVID-19 situation increased the turnover intention, especially among doctors and nurses (OR_nurse/midwife_ = 22.28 (2.78–41.25), *p* = 0.01; OR_doctors_ = 18.13 (2.22–2.27), *p* = 0.01) mediating the emotional pressure it was putting them under. Anxiety related to work-pressure and burnout especially were the main emotional predictors of turnover intention. The more severe the anxiety was, the more the professional considered quitting (OR_moderate_ = 18.96 (6.75–137.69), *p* = 0.005; OR_severe_ = 37.94 (2.46–107.40), *p* = 0.016). Only severe burnout, however, engendered such an intention among them (OR_severe_ = 13.05 (1.10–33.48), *p* = 0.000).

## 1. Introduction

### 1.1. Background

Healthcare professionals’ turnover is a worldwide rapid-growing human resource issue that is affecting healthcare systems. Their intention to quit (turnover) is associated with many factors that vary from mismatched expectations and workload to unsatisfying working conditions and other individual characteristics [1]. Research indicates that the emotional exhaustion and psychological health of healthcare professionals plays an important role in not only organizational outcomes but also performance and job attitudes [2].

The World Health Organization defines the worker’s psychological health as their ability to know their capabilities and understand their ability to overcome stress of working life without emotional destructions that may hinder the efficiency of their contributions [3]. Stress is a substantial problem for healthcare professionals [4]. It was posited that workers’ psychological health is in risk of deterioration if they work under stress for a long period of time [5]. Emotional exhaustion and turmoil led to serious problems related to their task accomplishments and job efficacy in many situations [6]. People’s emotional needs take a wider range when they are under pressuring circumstances and their emotional reactions become out of control. This emotional pressure takes on a larger scale when people are dealing with life-threatening events and disasters [7].

In pandemics, healthcare providers have the burden of being the crucial points of references for the patients and the citizens whenever they need to know more about the situation and the necessary measures to cope with the crisis, which may add a burden to the providers’ overwhelming task load. Impacts of responsibility burden are an issue that many healthcare professionals faced during the COVID-19 pandemic, and need to be urgently addressed [8].

Although COVID-19 has distressed the lives of millions of people since its first appearance in China in 2019, healthcare systems of developing countries, particularly, were the most impacted [9]. According to the Turkish Ministry of Health, COVID-19 was first detected in Turkey on 10 March 2020. By October 2021, a total of 50 thousand people had died, with a total of 6 million cases in Turkey [10]. As a result of this high demand, the national healthcare system was overburdened. In addition to the shortage in healthcare providers that the country has witnessed over the last years [11], the capacity of the hospitals was exceeded, and the providers’ workload became unmanageable.

The COVID-19 pandemic forced doctors to work under stressful situations, as they were facing the threat of death and contagion and taking responsibility for saving the lives of their patients with insufficient resources. In addition, many studies have investigated emotional exhaustion as a predictor of turnover in healthcare professionals in normal settings [2,12]. As in pandemic situations more healthcare resources are needed, it remains of great importance that we prevent active professionals from quitting their jobs due to the COVID-19 pandemic, and also in future crises that can harm the healthcare system. For these reasons, we investigated, in this study, psychological health as a predictor of intention to leave the job among healthcare professionals who were actively working during the COVID-19 pandemic.

### 1.2. Literature Review

Since the introduction of specific diagnostic criteria for mental disorders in the 1970s, the number of large-scale mental health surveys that provide population-level estimates of common mental disorders has exploded tremendously [13]. Literature describing mental health problems, such as depression, burnout, and anxiety, are highly prevalent around the world [14].

A state of depression can result in mood swings, pervasive sadness or disinterest, trouble concentrating, slowing down psychomotor function, a loss of energy, concentration problems, negative thoughts, loss of perspective on truth and reality, sleep disturbances, and suicidal ideas and behaviors [15]. There are many measures that quantify depression severity, such as the Zung Depression Rating Scale, Beck Depression Inventory (BDI) first (BDI I) [16], BDI second (BDI-II), and Beck Depression Inventory for Primary Care (BDI-PC) [17]. The Patient Health Questionnaire (PHQ) is also an instrument that evaluates depressive and other mental disorders from a criteria-based perspective. With only nine items, the PHQ is comparable in sensitivity and specificity to the other, longer measures [18]. Screening measures for depression do not diagnose the condition, but they do provide an indication of symptom severity, and assess their severity over a certain period of time (e.g., the last 7 to 14 days). A higher score consistently indicates more severe symptoms, despite each measure having its own scoring system. Depression symptoms are considered significant at a statistically predetermined cutoff score for all measures. Scores for some measures are divided into symptom severity categories.

Anxiety is one of the feelings that alerts individuals to anything harmful to worry about. With this alert mechanism, people evaluate the potentials of life-threatening situations and react to them in an appropriate and protective manner. There are several psychological symptoms of anxiety, including irritability, difficulty concentrating, and depression [19]. Physical symptoms include heart palpitations, sweating, tension and pain, heavy breathing, fainting, indigestion, stomach aches, sickness, and diarrhea; in acute cases, individuals have reported feeling as though they were dying [19]. Extreme forms of anxiety include not being able to rest or sleep well, becoming obsessive of thoughts that interfere with normal living, suppressing a job, or ruining relationships [19].

Anxiety is measured through many scales and tools. One of them is the BAI, known as the Beck Anxiety Inventory. It focuses on somatic symptoms of anxiety that were developed as a measure adept at discriminating between anxiety and depression [20]. Another scale is the Hospital Anxiety and Depression Scale Anxiety (HADS-A), which is a brief measure of generalized symptoms of anxiety and fear [21]. The State Trait Inventory (STAI), is also used to self-report the presence and severity of current symptoms of anxiety [22], and was developed in 1970 by Spielberger [23].

The term burnout, which has become commonly used today, originated with Freudenberg [24] in 1974 to describe emotional exhaustion experienced by workers in the public sector. There has been a strong focus on burnout among professionals who provide direct services to people, recognizing the special pressures that arise from working with difficult clients who are needy, demanding, and often troubled [25]. Burnout could be experienced when workers are faced with extreme stressors in the workplace and reply with generated behavior in response to those stressors [26]. Many measures are developed to measure burnout, such as the Maslach Burnout Inventory [27], Burnout Measure [28], Psychologists Burnout Inventory [29], Oldenburg Burnout Inventory [30], and Copenhagen Burnout Inventory [31].

Turnover is a deliberate separation from an organization by an individual [32]. It happens due to organizational problems, working conditions, and psychological issues [33]. Moreover, these factors might interact with each other to alter workers’ negative thoughts and emotions towards organizations. Krausz et al. (1995) argue that intention to leave is a better indicator of turnover [34], and some researchers found that intention to leave is statistically related to turnover [35].

## 2. Materials and Methods

### 2.1. Instrumentation

**Figure 1:**
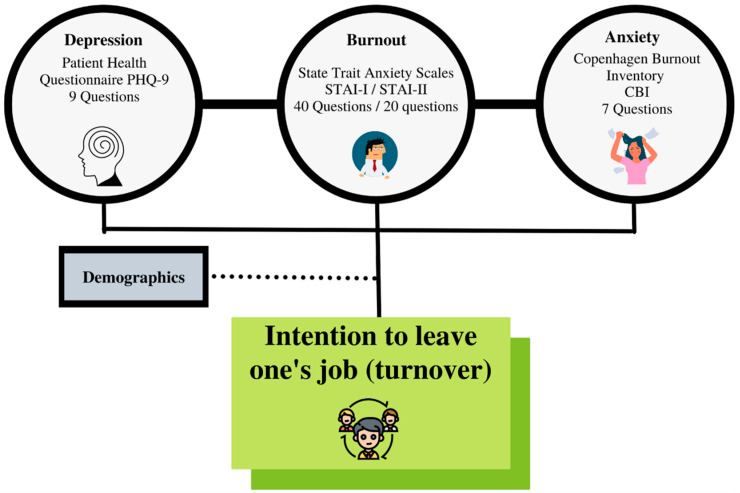
Framework of turnover possible predictors.

The study employed a survey questionnaire adopted from various scales. All survey questions were modified and adapted to fit the context of our research. For this study, the survey was designed to assess healthcare workers’ work health, work stress, interpersonal conflict, and intent to leave work. It also recorded respondents’ demographic information, such as gender, age, education level, the position of the worker, and their department. The Depression scale, the anxiety scales, the burnout scale, and the Turnover scale were, respectively, developed by Kroenke et al. [18], Spielberger et al. [23], Kristensen et al. [31], and Walsh et al. [36]. Anxiety scales are STAI-I OR S-Anxiety and STAI-II or T-Anxiety. The responses for the S-Anxiety scale assess intensity of current feelings “at the moment of the answer”, and those of the T-Anxiety assess the frequency of the feelings “in general”. Figure 1 summarizes the variables used with the scales’ names considered.

### 2.2. Study Design

The study was approved by the internal review board of Gazi Yaşargil Education and Research Hospital’s Ethics Committee (IRB ID 677). Surveys of 75-item questions (without considering demographic questions) were designed. Our data collection was completed in 2020 between March and April. A number (*n* = 700) of paper-based surveys were distributed to the healthcare workers’ offices. The research team collected the completed survey from the office, and we had almost a 50% response rate. A number (*n* = 344) of responses were considered from doctors, nurses, midwifes, health technicians, and other staff. Our data collection occurred in a public pandemic hospital in the Diyarbakir province of Turkey, which was secluded for infected patients. From March 2020 to March 2021, the hospital received (*n* = 87,355) positive COVID cases. A number (*n* = 1060) of health workers tested positive during this year, and (*n* = 3) of them died.

### 2.3. Statistical Data Analysis

First, descriptive statistics were conducted to check the distribution of the emotional health of the workers among the different demographic subgroups. Second, a chi-square test was run to calculate the significance of the correlation between the dependent variable of our study (outcome: intention to leave) and the demographical characteristics of the population. Then, by adjusting the model to the significantly correlated variables, we conducted a multivariable logistic regression analysis to check the impact of the independent variables of our models (predictors: burnout, depression, anxieties) on the dependent variable (intention to leave). *p* value of (<0.1) was considered as significant, as it indicates a positive correlation. All data cleaning and analyses were performed using Python 3.7 (The Python Software Foundation (PSF), Hoboken, United States of America).

## 3. Results

Among the respondents, 18.49% were registered doctors, 29.41% were registered nurses or midwifes, and the rest were other health staff, as mentioned in Table 1. A majority of 42.02% worked in emergency rooms (ER), and 59.24% of them were young (less than 36 years old). Looking at the distribution of the emotions, despite the majority of the respondents reporting low depression, (PHQ-9_low_ = 51.26%), a majority of them reported severe anxiety and high burnout level (ANXIETY1_severe_ = 74.79%, ANXIETY2_severe_ = 56.72%, Burnout_severe_ = 53.36%). Table 1 summarizes all the distributions. 

Based on the Chi-square test run, we found that only the job position of the respondents and the departments that they worked in correlated with their intention to leave their job, as mentioned in Table 2.

Thus, we adjusted our logistic multivariable model to the job position and department of the respondents. All results were summarized in Table 3. Anxiety 1 score, burnout, and job position were significant predictors of the employees’ intention to leave their job.

**Figure 2:**
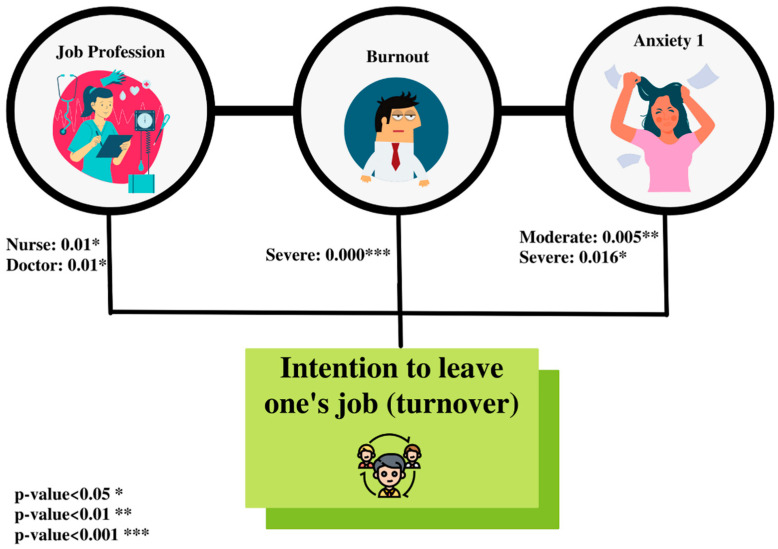
Predictors of turnover intention.

Adjusted for demographics, our model showed that the intense anxiety of healthcare workers is significantly correlated with their intention to leave their jobs. The more anxious they were, the more turnover intention became important (OR_moderate_= 18.96 (6.75–137.69), *p* = 0.005; OR_severe_ = 37.94 (2.46–107.40), *p* = 0.016). However, general anxiety STAI-II was not correlated with the turnover. Another cause of the turnover intention was severe burnout (OR_severe_ = 13.05 (1.10–33.48), *p* < 0.001). In addition, doctors and nurses were significantly more likely to intend to leave their jobs. Nurses and doctors were more likely to have the intention to quit than other healthcare staff (OR_nurse/midwife_ = 22.28 (2.78–41.25), *p* = 0.01; OR_doctors_ = 18.13 (2.22–2.27), *p* = 0.01). Figure 2 summarizes the significant results of this study.

## 4. Discussion

This study is one of the first conducted during the COVID-19 peak, in one of the most pandemic-impacted healthcare systems in developing countries. Our study showed that the psychological health of healthcare professionals caused by the pressure of the pandemic were significantly positively correlated with the workers intention to leave their jobs. We found that the more healthcare professionals had work-related anxiety and burnout, the more they intended to quit. However, their depression and general anxiety level were not among the predictors of the turnover. This proves that the COVID-19 situation is increasing the turnover intention especially among doctors and nurses, who are mediating the emotional pressure it is putting them under.

COVID-19 is an emerging infectious disease threat that requires more than regular healthcare professionals to stay on regular duty. Pandemics may require longer hours (and correspondingly higher levels of exposure to the virus), quarantines, and assignments outside a doctor’s specialty and a nurse’s set of skills [37,38]. This adds moral and physical stress to them [39], which comes from the workload that is added to their capacity, the uncertainty that comes with the changes in regulations, the tough decisions they have to make, and many other causes. COVID-19 specifically adds more difficulty to the situation by putting the healthcare professionals at risk of death and contamination [39]. Another recent study from Turkey also showed that healthcare providers who work in pandemic hospitals had significantly higher job stress compared to healthcare workers who worked in non-pandemic hospitals [20].

Even though healthcare professionals were considered to be the “heroes” of the pandemic, calling them heroes does not protect them from psychological trauma and job stress. Ana Delgado, a nurse, midwife, and clinical professor in San Francisco, California said:

“*I want to be recognized for my hard work, but I feel like it will swing back to the other side, to mistrust and lack of support. If you think about traditional societies, the position of a healer is very respected, but it is also accountable to the community*.”

[40]

Work burnout and anxiety have always been highlighted as causes of emotional exhaustion that can hinder healthcare providers’ performance [41]. During the pandemic, front-line caregivers have faced hard decisions to make [42] and have lived situations of injustice due to the pandemic that have made them feel powerless, all of which has been associated with the burnout they have felt. COVID-19-related anxiety and burnout not only made it hard for health workers to practice their jobs, but also exhausted their emotions and made them doubt their ability to take care of their patients and to save them which may explain why these emotions were highly correlated with the healthcare professionals’ intention to leave their jobs. This may explain why, in our study, professionals who had severe work-related anxiety and burnout were more likely to intend to quit their jobs. However, the general anxiety or depression that a professional may have, or that is related to other causes, were not correlated with the turnover intention. Furthermore, doctors and nurses faced the most challenging situations as decision-makers during the pandemic. The critical situation that they were put in on the front-line with more workload was overwhelming enough that they would want to quit their jobs more than other professionals. This correlates with the findings of our study.

### Implications

With a system already suffering from scarce human resources to provide care [43], the last thing hospitals need during the pandemic is to lose their doctors, nurses, and staff when they need them the most. Thus, more attention towards healthcare professionals’ health and mental wellbeing is needed. Hospital personnel will be stressed and overwhelmed by the day-to-day challenges, and it is the role to the managers to provide emotional support and encourage them to practice self-care. In addition, organizing the flow of information between the different providers may help them better use the feedback provided to improve their work, embedding all the changes into protocols in real-time. More meetings with managers of the organization can help secure each personnel’s role in a better way. Citizens also should be sensitized to the importance of considering their interactions with their caregivers as a partnership, respecting their sacrifices and taking mutual responsibilities of the occurring outcomes to reduce both sides’ anxiety and frustration.

**Figure 3:**
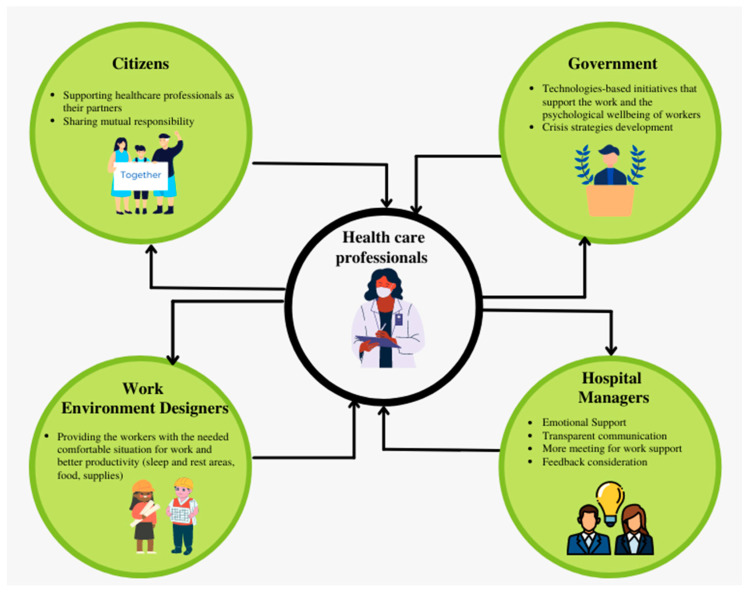
Crisis management recommendations to support healthcare professionals’ self-control.

Furthermore, the environment of work has an important impact on the performance of workers, especially in healthcare [44]. For this reason, in order to control the anxiety, stress and alleviate workload-related burnout, better work environments should be provided to the professionals working in pandemic facilities, with more ergonomic design (food, sleeping areas, rest spaces, etc.). Finally, technology-based initiatives should be used to protect doctors and alleviate their health-related risk. For example, telemedicine or telehealth can effectively minimize virus spread, use healthcare professionals’ time efficiently by optimizing their workload, and support their mental health and emotional wellbeing. These factors are summarized in the framework suggested in Figure 3.

These findings are related to a certain crisis period. It is important to consider them while developing strategies that aim to cope with disasters. However, we should also check in future research whether the same emotions’ impacts on turnover can be generalized to normal settings to improve working environments. In future research, we can also compare developing and developed countries, and the strategies they use to deal with the pandemic in order to understand how each system can take inspiration from the other to create a more balanced healthcare system worldwide.

Some limitations of our study should be acknowledged. We only included participants from one hospital in Turkey, which makes it difficult to generalize the results, although we do know that the findings and insights are supportive of healthcare systems worldwide. In addition, we did not consider a control group, which we should consider in future studies. We consider using data that is nationally representative to confirm our findings in studies [45].

## 5. Conclusions

The COVID-19 outbreak marks a vital moment where healthcare systems could be revolutionized. The front-line providers have so far been the heroes that have stood by their patients trying to save them with the resources given to them. The increased workload and responsibilities have added an emotional load on these professionals. Based on our findings from this study, run in a pandemic hospital in a developing country, anxiety and burnout caused the providers to consider quitting their jobs. This was especially noticed among nurses and doctors, as they have the most stressful jobs during disasters. These findings may concern many other hospitals from developing and developed countries too. Thus, attention needs to be given to the healthcare professionals’ emotional wellbeing to support them in their jobs and to avoid losing a scarce resource when we much need it. 

## Figures and Tables

**Table 1:** Descriptive statistics of emotional health distribution of the respondents.

Predictors		PHQ9			ANXIETY1			ANXIETY2			Burnout			Total %
		L ^1^	M ^2^	S ^3^	L	M	S	L	M	S	L	M	S	
**Sex**	**Male**	17.21%	4.84%	25.93%	24.00%	17.14%	14.61%	16.28%	11.67%	17.78%	17.50%	14.08%	16.54%	15.97%
	**Female**	82.79%	95.16%	74.07%	76.00%	82.86%	85.39%	83.72%	88.33%	82.22%	82.50%	85.92%	83.46%	84.03%
**Education**	**Middle School**	62.30%	69.35%	75.93%	76.00%	62.86%	66.85%	79.07%	60.00%	66.67%	52.50%	56.34%	77.95%	67.23%
	**High School**	20.49%	14.52%	5.56%	12.00%	11.43%	16.85%	9.30%	18.33%	16.30%	30.00%	16.90%	10.24%	15.55%
	**University**	4.10%	14.52%	12.96%	12.00%	5.71%	8.99%	4.65%	5.00%	11.85%	12.50%	14.08%	4.72%	8.82%
	**Doctorate**	13.11%	1.61%	5.56%	0.00%	20.00%	7.30%	6.98%	16.67%	5.19%	5.00%	12.68%	7.09%	8.40%
**Job Position**	**Health Staff**	56.56%	50.00%	44.44%	52.00%	48.57%	52.81%	46.51%	51.67%	54.07%	67.50%	57.75%	44.09%	52.10%
	**Nurse or Midwife**	18.85%	40.32%	40.74%	28.00%	22.86%	30.90%	30.23%	21.67%	32.59%	17.50%	19.72%	38.58%	29.41%
	**Doctor**	24.59%	9.68%	14.81%	20.00%	28.57%	16.29%	23.26%	26.67%	13.33%	15.00%	22.54%	17.32%	18.49%
**Department**	**Other**	36.89%	16.13%	20.37%	48.00%	31.43%	24.16%	30.23%	30.00%	25.93%	42.50%	29.58%	22.05%	27.73%
	**ER**	41.80%	43.55%	40.74%	20.00%	42.86%	44.94%	41.86%	45.00%	40.74%	27.50%	45.07%	44.88%	42.02%
	**Inpatient Unit**	18.03%	27.42%	29.63%	24.00%	20.00%	23.60%	23.26%	18.33%	25.19%	20.00%	18.31%	26.77%	23.11%
	**ICU**	3.28%	12.90%	9.26%	8.00%	5.71%	7.30%	4.65%	6.67%	8.15%	10.00%	7.04%	6.30%	7.14%
**Age**	**Young Adults**	57.38%	58.06%	64.81%	64.00%	62.86%	57.87%	60.47%	61.67%	57.78%	60.00%	47.89%	65.35%	59.24%
	**Middle Age or More**	42.62%	41.94%	35.19%	36.00%	37.14%	42.13%	39.53%	38.33%	42.22%	40.00%	52.11%	34.65%	40.76%
**Total %**		51.26%	26.05%	22.69%	10.50%	14.71%	74.79%	18.07%	25.21%	56.72%	16.81%	29.83%	53.36%	51.26%

^1^ L: Mild or Low, ^2^ M: Moderate, ^3^ S: High or Severe.

**Table 2:** Correlation between the demographic variables and the intention to leave.

Predictors		People Who Have the Intention to Leave (%)	*p*-Value
**Sex**	**Male**	51.52%	0.542
	**Female**	48.48%	
**Education**	**Middle School**	75.76%	0.233
	**High School**	10.61%	
	**University**	1.52%	
	**Doctorate**	12.12%	
**Job Position**	**Health Staff**	39.39%	0.0517 *
	**Nurse or Midwife**	37.88%	
	**Doctor**	22.73%	
**Department**	**Other**	19.70%	0.0423 *
	**ER**	45.45%	
	**Inpatient Unit**	28.79%	
	**ICU**	6.06%	
**Age**	**Young Adults**	86.36%	0.594
	**Middle Age or More**	13.64%	

* *p* < 0.05.

**Table 3:** Results of the multivariable logistic regression of the intention to leave jobs.

Predictors		Odds Ratios	*p*-Value
**PHQ9**	**Low**	**NA**	
	**Moderate**	4.79 (0.90–33.06)	**0.08**
	**Severe**	12.53 (1.18–368.37)	**0.06**
**Anxiety 1**	**Low**	**NA**	
	**Moderate**	18.96 (6.75–137.69)	0.005 **
	**Severe**	37.94 (2.46–107.40)	0.016 *
**Anxiety 2**	**Low**	**NA**	
	**Moderate**	0.16 (0.008–1.88)	**0.17**
	**Severe**	0.14 (0.008–1.43)	**0.12**
**Burnout**	**Low**	**NA**	
	**Moderate**	9.97 (1.03–164.48)	**0.06**
	**Severe**	13.05 (1.10–33.48)	0.000 ***
**Department**	**Other**	**NA**	
	**ER**	3.22 (0.56–22.40)	**0.2**
	**Inpatient Unit**	2.83 (0.39–23.74)	**0.3**
	**ICU**	3.30 (0.06–576.09)	**0.57**
**Job Position**	**Health staff**	**NA**	
	**Nurse/midwife**	22.28 (2.78–41.25)	0.01 *
	**Doctor**	18.13 (2.22–25.27)	0.01 *

*p < 0.05, ** *p* < 0.01, *** *p* < 0.001.

## Data Availability

The data presented in this study are available on request from the corresponding author.
